# Influence of Pre- and Post-Contouring Strategies to Downskin Sloped Surfaces in Laser Powder-Bed Fusion (L-PBF) Additive Manufacturing

**DOI:** 10.3390/ma17112639

**Published:** 2024-05-30

**Authors:** Nismath Valiyakath Vadakkan Habeeb, Rabiul Islam, Kevin Chou

**Affiliations:** Department of Industrial Engineering, University of Louisville, Louisville, KY 40292, USAkevin.chou@louisville.edu (K.C.)

**Keywords:** downskin surfaces, surface roughness, laser powder-bed fusion, contouring strategies

## Abstract

Among various metal additive manufacturing (AM) technologies, L-PBF is known for fabricating intricate components. However, due to step edges and powder particle attachments, attaining a good surface finish is challenging, especially on downskin surfaces. Contour scanning has potential to improve surface quality because such scanning may dominate the surface formation of sloped features. This study evaluates the effects of pre- and post-contouring strategies on the sloped downskin surfaces fabricated using a commercial L-PBF system with Ti6Al4V powder. L-PBF parts printed at inclination angles 30°, 45° and 60° were investigated. A double-contouring approach with varying processing conditions was employed and surface characteristics were analyzed using data acquired by white light interferometry. The average surface roughness, S_a_, surface skewness, S_sk_, and percentage area of powder particles attached onto surfaces were statistically evaluated. The lowest S_a_ obtained for pre- and post-contoured samples is 14.08 µm and 18.88 µm, respectively. For both strategies, the combination of a low laser power and a high scan speed on the interface of downskin surface and underneath powder results in smoother surfaces. However, while comparing both strategies, pre-contouring gives better surface finish for samples built at similar processing conditions, with a difference of nearly 5 µm in S_a_.

## 1. Introduction

Laser Powder Bed Fusion (L-PBF) is a widely adopted additive manufacturing process used to fabricate complex metallic parts. The process selectively melts a layer of metal powder by laser scanning across the powder bed. Subsequently, the powder bed lowers down one layer thickness, and a new layer of metal powder is spread on top of the melted layer. Again, the laser beam scans along the predetermined path, building the part layer by layer [1]. The L-PBF process excels in producing complex geometries with single-step fabrication and reducing the weight of a component while meeting the mechanical requirements. These unique advantages have led to the process being utilized in various industries like biomedical, automotive, and tooling, but most notably the aerospace sector [2]. 

Despite advances in the L-PBF process, it still faces challenges that hinder its growth in many industries. Due to lower dimensional accuracy and process repeatability compared to conventional methods, as-built parts require additional post-processing like polishing, coating, and chemical treatments. Moreover, mechanical properties, particularly fatigue strength, are constrained by factors such as porosity and surface roughness [3]. 

One of the benefits of L-PBF comes from being able to additively build parts without the need for support structures. However, parts built with unsupported overhangs exhibit higher surface roughness, and the detrimental effect of surface roughness on fatigue performance is well examined. Sanaei and Fatemi have found that surface roughness significantly impacts fatigue performance, even in the presence of internal defects and varying microstructures [4]. So, it is of high interest to explore ways to minimize surface roughness at low overhang angles. Also, surface roughness is an important consideration for L-PBF due to its effect on assemblability, which is defined as a component’s capability to assemble smoothly with a mating component and depends on the geometric tolerances of mating parts [5]. In addition to considerations of friction, L-PBF-produced parts with high surface roughness require postprocessing steps, resulting in additional costs and time [6]. Furthermore, some applications in the bio-medical sector require tailored surface roughness, so it is imperative that we establish the effect of process parameters on surface roughness [7].

Among many ways to quantify and characterize surface roughness (S_a_, S_sk_, S_ku_, S_t_, S_q_), the S_a_ value, which is defined as average height of a surface, is the most used as the other parameters have strong correlation to S_a_ value and overall provides a better estimate of surface roughness [8]. To measure the roughness, contact stylus profilometry remains a staple for precise topography measurement at a microscale level. However, these contact-based methods are sometimes inadequate in measuring a surface area instead of along a line [9]. Scanning Electron Microscopy (SEM) imaging allows for detailed visualization of surface features and provides valuable qualitative information about surface texture, morphology, and defects [10,11]. However, for precise surface generation with numerical modelling capabilities, X-ray Computed Tomography (XCT) and Scanning White Light Interferometry (SWLI) work well [11,12]. 

When examining surface roughness in parts built with unsupported overhangs, the surface facing downward (downskin) is found to have higher surface roughness than upward facing (upskin), vertical (sideskin), and horizontal surfaces [13]. However, the causes and mechanisms of surface formation are different for upskin and downskin. Hence, the parameters affect both surfaces differently. Two primary contributors to upskin surface roughness are step edges and the agglomeration of partially sintered particles [14,15]. Strano et al. [16] examined the relationship between overhang sloping angles and upskin surface roughness and found that for lower sloping angles, the staircase effect was the main cause of surface roughness, while at higher sloping angles, partially sintered particles attached to step edges was the dominant factor.

Downskin surface roughness is mostly due to the phenomena called dross formation which is caused by the difference in heat transfer rate between solidified metal and metal powder. As the laser irradiates a region with solid support, the heat dissipation is fast due to high thermal conductivity of solid metal. Conversely, when the laser hits a powder-supported region, the heat dissipation is slower and leads to larger melt pools. The enlarged melt pool then sinks into the powder bed due to gravity and capillary forces resulting in dross formation [17,18,19]. The sagging melt pool produces a larger area for partially melted powder particle attachment, thus contributing to higher surface roughness. 

A number of processes, design, and metallurgical factors affect downskin surface quality. Scan strategy (contouring, hatch spacing, skywriting) and process parameters (scan speed, laser power, hatch spacing, layer thickness), among others, impact downskin surface roughness. Tian et al. examined the influence of process parameters on surface roughness of Hastelloy-X parts made by L-PBF [20]. They found that low laser power and high scan speed produce low downskin roughness. Fox et al. studied the downskin of stainless steel-GP1 parts at various overhang angles and observed overall surface roughness increase with decreasing inclination angle. Also, they found that partially melted particles dominate the downskin surface at a very low (0.06 J/mm) linear energy density, and at a higher (0.28 J/mm) linear energy density, along with partially melted powder, there are solidified melt tracks and deformed materials by the recoater on the surface [21]. Snyder and Thole identified melt pool depth as the primary predictor of downskin surface roughness and found that contour parameters have significant impact for downskin surface [22]. Ullah et al. identified sub-surface pores as another factor that affects the downskin surface quality of AM parts. The sub-surface porosity was found to increase with the inclination angle of the overhang surfaces. They also found that the contour scanning strategy can affect the downskin surface roughness as well as sub-surface porosity to a great extent [23].

Although several studies have shown that contouring of the scan region is an effective way to reduce the surface roughness of L-PBF parts, a comprehensive analysis with various contouring methods and a wide range of contouring parameters still cannot be found. This study aims to study the effects of two different contouring strategies, namely pre- and post- contouring on the downskin surface roughness of sloped L-PBF parts. A wide range of contouring parameters including laser power and scan speed were taken into account, and the effects of these variables on downskin surface roughness were analyzed qualitatively and quantitatively from surface measurement results.

## 2. Methodology

### 2.1. Sample Design and Fabrication

**Figure 1:**
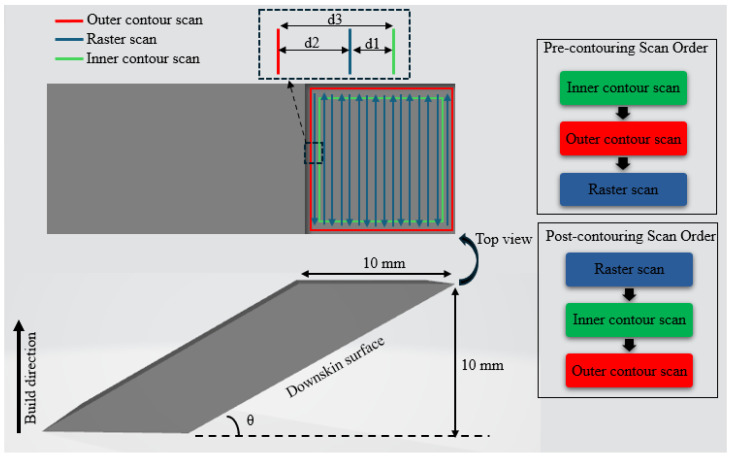
Design of the sloped sample with illustration of the contour scan strategy and scan order.

The design of experiment (DOE) was created with two different contouring strategies, namely pre-contouring and post-contouring. To ensure adequate remelting of the contouring region, a double-contouring technique rather than a single contour scan was adopted in both cases. An inner contour scan shifted inside the raster scan region at an offset distance, d1, and an outer contour scan shifted towards the outside of the raster scan at offset distance, d2, were implemented. Along with laser power (P), scan speed (V), and the inclination angle (θ) of the downskin surfaces, the total offset distance, d3 (total distance between inner and outer contour scans, d3 = d1 + d2), was taken as a parameter while designing the experiment. The DOE with both strategies is explained in Section 2.1.1 and Section 2.1.2. The scan strategy and order are illustrated in Figure 1, along with the sample design.

**Figure 2:**
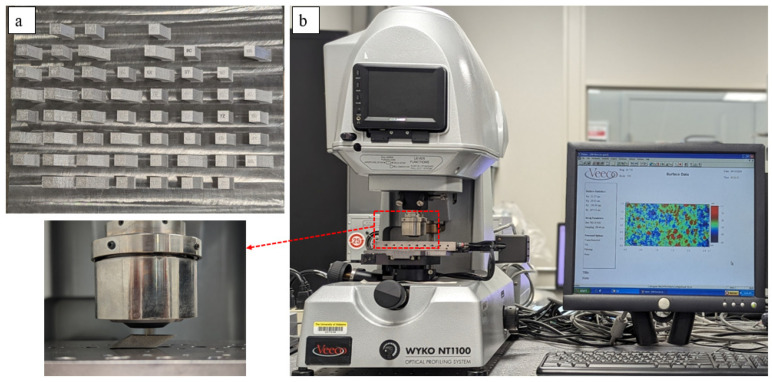
(**a**) Fabricated sloped samples on the build plate. (**b**) White Light Interferometer (WLI) used for the downskin surface measurements.

The sloped samples with width and height of 10 mm, at inclination angles of 30°, 45°, and 60°, were designed as shown in Figure 1. An EOS M270 laser powder-bed fusion system and Ti6Al4V powder were used for fabrication of the sloped samples. A powder layer thickness of 30 µm, beam diameter of 100 µm, and hatch spacing of 100 µm were applied throughout the fabrication process. In addition, default raster scan parameters of 170 W laser power and 1250 mm/s scanning speed were used for all samples, which are the EOS machine recommended process conditions for Ti6Al4V printing at the selected beam diameter and layer thickness. The raster scans were rotated through 90° in each layer. The build plate design, slicing of STL files, and process parameter setup were performed using various software such as Materialize Magics 24.0, RP Tools 6.0, and EOS PSW V3.5. Three replicates of each sample were fabricated (Figure 2a), and the samples were placed randomly on the build plates to avoid the influence of location-dependent effects.

#### 2.1.1. Pre-Contouring Strategy

In this strategy, the inner contour scan will be followed by the outer contour scan at distance d3, and then the raster scans. According to a study on the effect of contour scan sequence on the surface roughness of a L-PBF copper alloy part, the pre-contouring resulted in lower roughness on the vertical surfaces. The presence of powder provides better energy absorption during pre-contouring, which improves the melt behavior and hence the surface quality [24]. However, the effect of double contouring prior to raster scans on the inclined downskin surfaces has not yet been reported. 

The design of the experiment (DOE) with pre-contouring strategy is listed in Table 1. For this experiment, a fractional factorial experiment with laser power, P, scan speed, V, inclination angle, θ, and offset distance, d3, was implemented. Each variable has three factor levels set up by taking the default machine recommended contour scan parameters (P = 150 W, V = 1250 mm/s, d3 = 20 µm) as the pivot parameter. The default offset distance in the machine setup is 20 µm. Other values were selected in reference to the previous study using post-contouring strategy, where the offset distance, d3, has shown a significant effect on the surface roughness [25].

#### 2.1.2. Post-Contouring Strategy

For the post-contouring strategy experiment, the process variables are different from that of pre-contouring strategy. The downskin surface quality is highly dependent on the melt pool characteristics and hence the process parameters that dictate the surface formation. The high energy density of the laser beam can help in remelting the edges of scanning region. However, it also results in a larger melt pool, causing higher chances of particle attachment on the downskin surfaces [17]. In order to maintain the remelting property and reduce powder attachment, a specific strategy with a high-linear-energy-density (LED) inner contour scan followed by a relatively low LED outer contour scan was incorporated. The lower LED helps in smoothening the interface between the molten region and the powder bed while reducing the surface area for powder particle attachment on the downskin surface. The offset distance between the contour scans was set up as 20 µm, for all cases, to focus on the effect of the scan strategy itself. The medium-LED case had the default parameters of 150 W laser power and 1250 mm/s scan speed. The low-LED and high-LED cases were selected relative to the default parameter. The scan speed was not varied for the individual samples; only the laser power was set differently for the inner and outer contours. The DOE is shown in Table 2.

### 2.2. Surface Measurements

The downskin surface roughness was measured using a white light interferometer (WYKO NT1100, Veeco Instruments. Inc., Plainview, NY, USA), shown in Figure 2b. A white light source in the machine is directed towards the reference mirror as well as the sample surface. The machine uses a vertical scanning interferometry (VSI) mode, where the surface at varying heights is scanned by moving the objective lens (50×) in a vertical direction (*Z*-axis). The interference fringes at each vertical position formed from the reflections of both reference mirror and sample surface were collected and used for the analysis. The back scan and scan length from the focus were set as 250 µm and 100 µm, respectively. A scanning area of 2.65 mm × 1.25 mm was set in the center of each sample surface by moving the stage in *X*- and *Y*-axes. The pixel resolution for the images obtained was 340 nm × 394 nm. The Vision v3.60 software was used for the WLI image analysis, and the Minitab 20.3 statistical software was used for the roughness data analysis.

## 3. Results

### 3.1. Pre-Contouring Strategy

**Figure 3:**
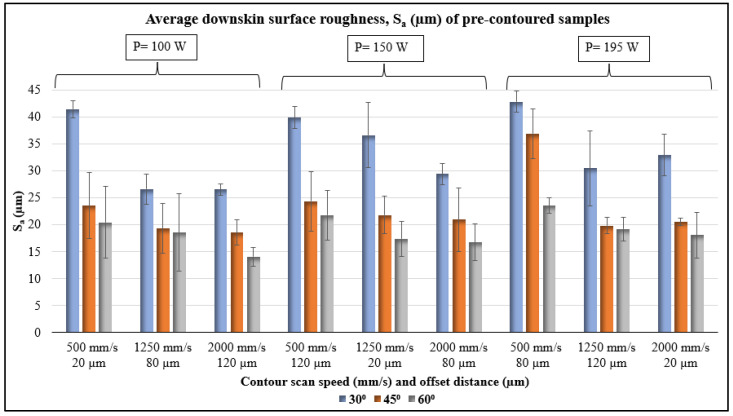
Average downskin surface roughness, S_a_, data of pre-contoured samples.

The average surface roughness, S_a_, obtained from the downskin surfaces of pre-contoured samples are plotted in Figure 3. The sample fabricated with a low-LED pre-contouring (P = 100 W, V = 2000 mm/s, LED = 0.05 J/mm) and inclination angle 60° had the lowest S_a_ value of 14.08 µm. The highest S_a_ value of 42.84 µm was obtained for the sample with highest LED pre-contouring (P = 195 W, V = 500 mm/s, LED = 0.39 J/mm) and inclination angle 30°. From the results, in general, the S_a_ value decreases with an increase in scan speed for a given laser power. Likewise, with an increase in laser power from 100 W to 195 W, there is a slight increase in the S_a_ value in most cases for all inclination angles. The results suggest a strong correlation of pre-contouring parameters and the downskin surface roughness. However, the statistical analysis using ANOVA was also performed correlate the effect of each variable to the downskin surface roughness more distinctively. 

**Figure 4:**
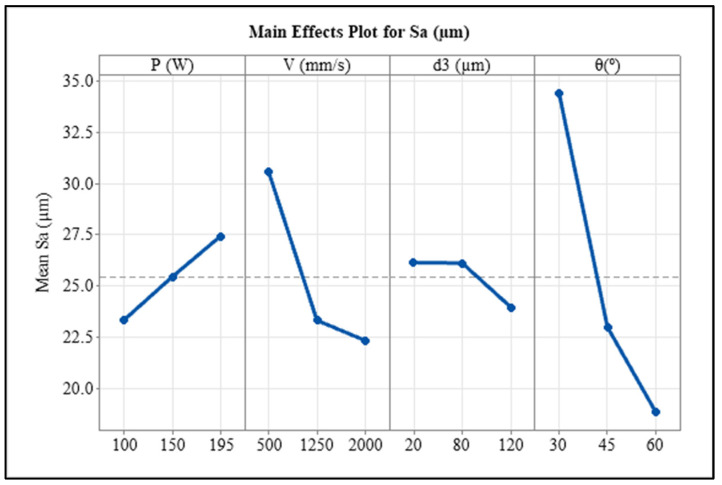
Main effect plot for S_a_ of pre-contoured samples.

Statistical analysis using ANOVA was performed for correlating the effect of each variable to the downskin surface roughness. The results are shown in Table 3 and the main effects plots for the four variables are in Figure 4. The *p*-values indicate a significant effect (*p*-value < 0.05) of pre-contouring laser power, scan speed, and inclination angle on the surface roughness at a 95% confidence interval. The offset distance has a high *p*-value and hence does not show an impact on S_a_ value. This might be because of the repeated remelting of the pre-contoured region by the raster scans. The percentage contribution of inclination angle to the S_a_ value (55.29%) is notably higher than all other variables. The scan speed has the second highest contribution (17.25%) to the average surface roughness. The literature also reports findings that are similar. The inclination angle and the scan speed were the vital parameters that determined the downskin surface roughness of AISI 316L L-PBF parts, according to Wang et al.’s investigation on the effects of laser power, scan speed, and hatch spacing on the downward-facing surfaces [26]. The main effect plot suggests similar trends as in Figure 3.

**Figure 5:**
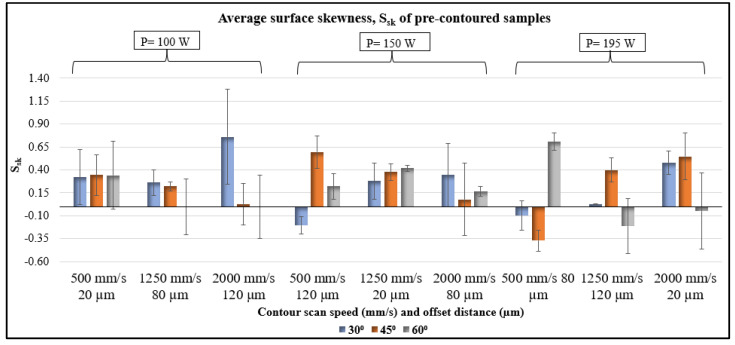
Average surface skewness of pre-contoured samples.

The average skewness data of the pre-contoured samples are shown in Figure 5. Skewness is a measure of the degree of variation of surface peaks and valleys from the mean plane. A zero skew (S_sk_ = 0) indicates a symmetrical plane while a positive (S_sk_ > 0) and negative skew (S_sk_ < 0) indicate variation of the surface above and below the mean plane, respectively. The skewness data obtained for the pre-contoured samples are in a range between −0.4 and 0.8, with no trend with respect to the processing parameters and inclination angle. In the case of the peaks and valleys formed due to step edges and the powder particle attachment corresponding to the LED used for pre-contouring, a trend relating the process variables and S_sk_ values is expected. This suggests that here, the skewness is not dependent entirely on the inclination angle of the downskin or the processing conditions but also on random powder particle attachment. The ANOVA results of S_sk_ values are tabulated in Table 4. The data validates the observation from Figure 5 as the *p*-value is larger than 0.05 for all variables. 

**Figure 6:**
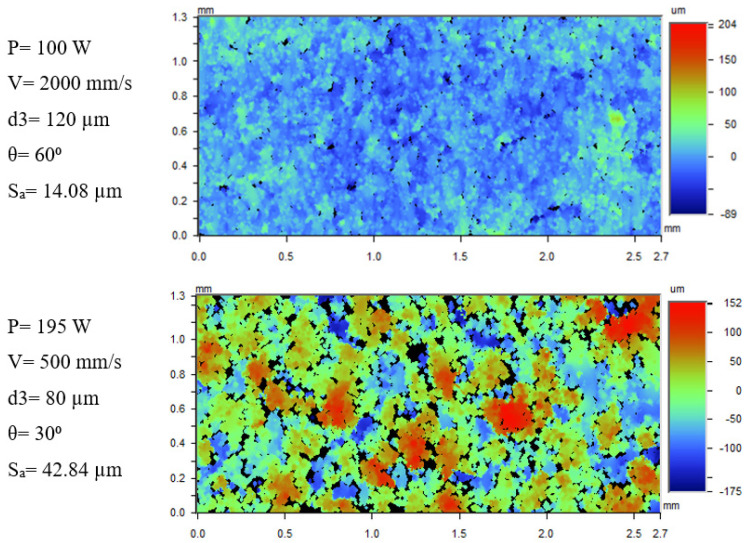
WLI images of pre-contoured samples with highest and lowest downskin surface roughness.

Figure 6 shows the WLI images of the samples with lowest and highest surface roughness. The 60°-inclined downskin surface built with low LED (0.05 J/mm) has no visible surface irregularities in comparison with the 30°-inclined surface at high LED (0.39 J/mm).

**Figure 7:**
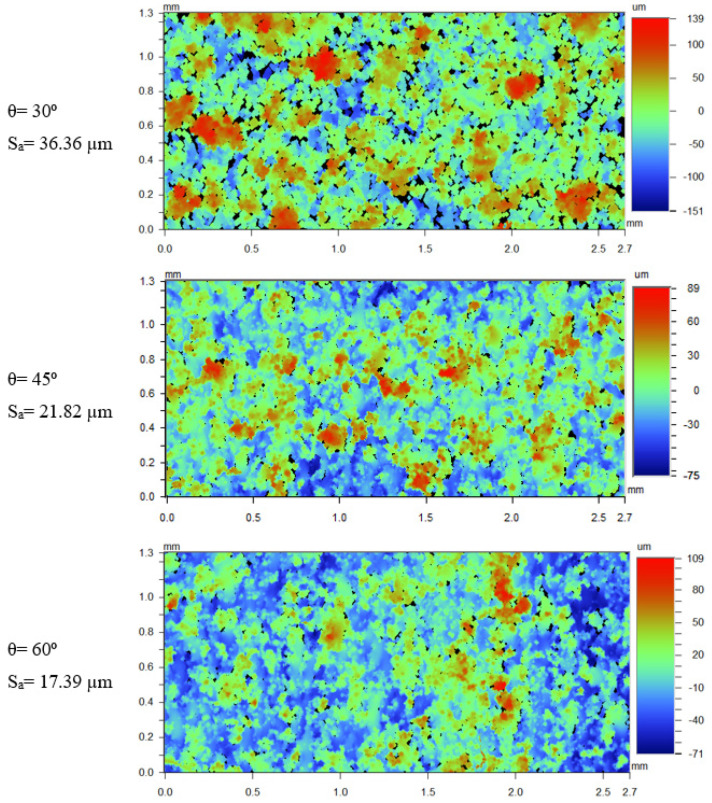
WLI images of samples built at parameters P = 150 W, V = 1250 mm/s, d3 = 20 µm, and different inclination angles.

The images of samples built with different inclination angles and at same processing parameters (P = 150 W, V = 1250 mm/s, d3 = 20 µm) are shown in Figure 7. The surface at 30° inclination seemingly has more powder particles attached compared to the 45°- and 60°-inclined surfaces.

**Figure 8:**
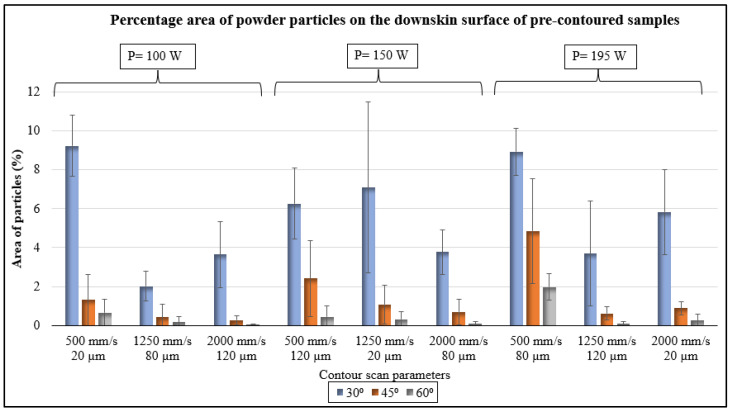
Percentage area of powder particles attached to the downskin surfaces of pre-contoured samples.

The percentage area covered by the powder particles on the WLI images was calculated by filtering the area that has Z-height above 70 µm using the Vision software v 3.60. The data (Figure 8) indeed confirms the analysis from the images, as the 30°-inclined samples shows a larger area covered by particles when compared to the 45°- and 60° samples.

### 3.2. Post-Contouring Strategy

**Figure 9:**
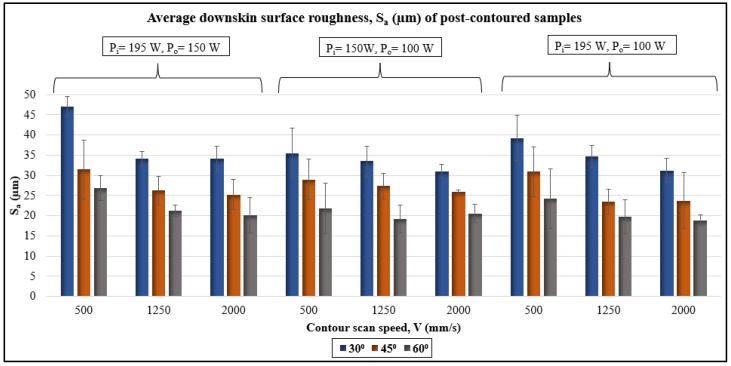
Average downskin surface roughness, S_a_, data of post-contoured samples.

The average downskin surface roughness, S_a_, data from the WLI measurements of post-contoured samples is plotted in Figure 9. The lowest S_a_ (18.88 µm) is for the sample built with inner contour laser power (P_i_) of 195 W, outer contour laser power, (P_o_) of 100 W, contour scan speed (V) of 2000 mm/s, and inclination angle (θ) of 60°. The highest S_a_ of 47.10 µm is for the sample built with P_i_ of 195 W, P_o_ of 150 W, V of 500 mm/s, and inclined at an angle 30°. In general, the average downskin surface roughness is found to be lower for the samples built at inclination angle, 60° compared to those with 30°. The medium-LED inner contour–low-LED outer contour case (P_i_ = 150 W, P_o_ = 100 W) has lower downskin surface roughness compared to the other two cases using high-LED inner contouring. The medium-LED inner contour case results in lower melt pool depth and width [25]. It helps in reducing the amount of powder particles becoming attached to the downskin surface due to smaller exposed areas of the melt pool.

**Figure 10:**
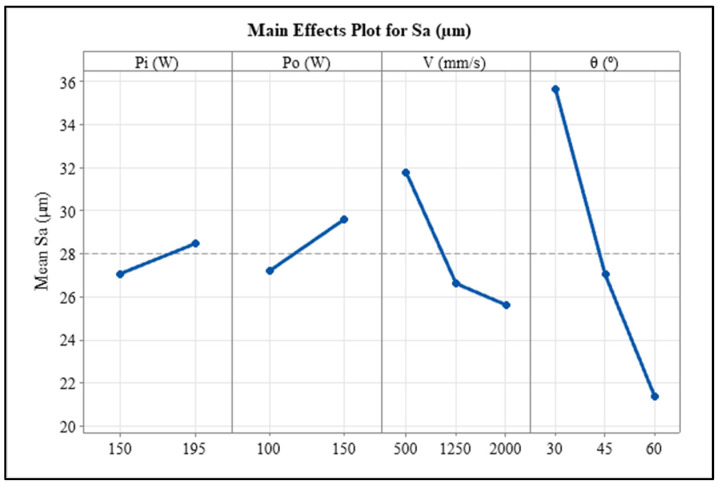
Main effects plot for downskin S_a_ of post-contoured samples.

Statistical analysis using ANOVA was also performed to understand the independent effects of variable parameters on the downskin surface roughness. The results are shown in Table 5 and the main effects plot is in Figure 10. The ANOVA table shows that other than inner contour scan power, all other variables are significantly affected the downskin surface roughness (*p*-value < 0.05) at a confidence interval of 95%. However, the percentage contribution of inclination angle is the highest, followed by that of scan speed, which could be the reason for the higher *p*-values for both laser power variables. The main effect plot also shows a slight difference between the two contour powers, whereas the scan speed and inclination angle affect the S_a_ value the most. The S_a_ value decreases with increases in scan speed because of the smaller melt pool reducing the chances of powder particle attachment. As the inclination angle increases from 30° to 60°, the surface roughness decreases considerably.

**Figure 11:**
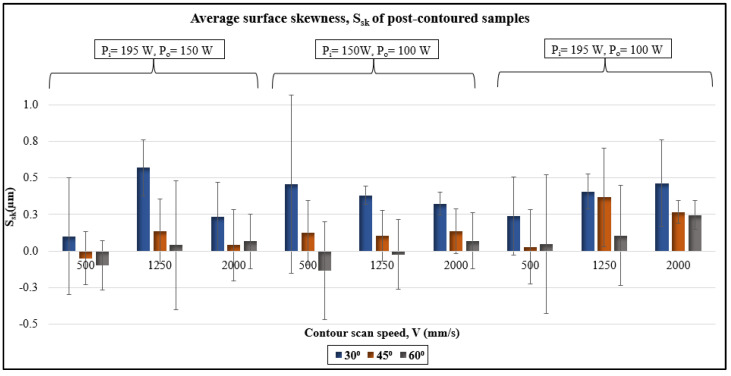
Average surface skewness data of downskin surfaces of post-contoured samples.

The average surface skewness data is plotted in Figure 11. No trend was observed corresponding to the process parameters used. Statistical analysis using ANOVA was performed and the results are shown in Table 6. The *p*-value > 0.05 for all variables except the inclination angle, and hence only inclination angle has significant effect on the skewness of the downskin surface. The variations are high on the 30°-inclined samples, whereas the skewness values are close to zero for the 60°-inclined samples. The skewness is hence affected by the randomly attached powder particles, which are determined by the surface’s inclination angle and are not due to the process-induced peaks and valleys.

**Figure 12:**
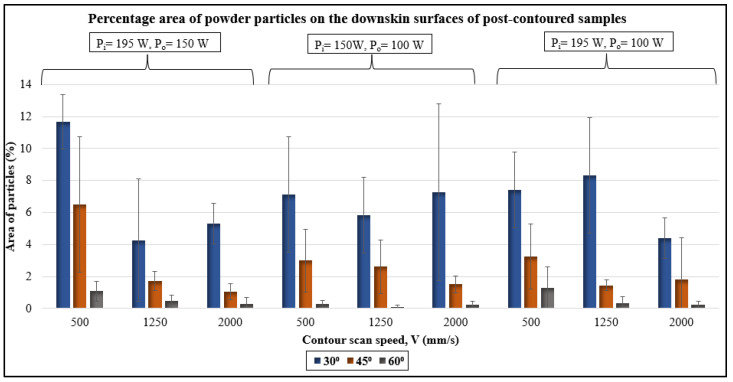
Percentage area of powder particles on the downskin surfaces of post-contoured samples.

The percentage area of particles was calculated from the collected data to validate the skewness results and is shown in Figure 12. The data shows that the 30°-inclined samples have a very large area covered with powder particles, and that could be the reason for the positive skewness. The data of powder particles present on the 30°-inclined downskin surfaces can also be directly correlated to their higher average surface roughness. The area of particles on 60°-inclined samples are comparatively very low (ranging from 0.1% to 1.28%), resulting in near-zero skewness and a lower surface roughness.

**Figure 13:**
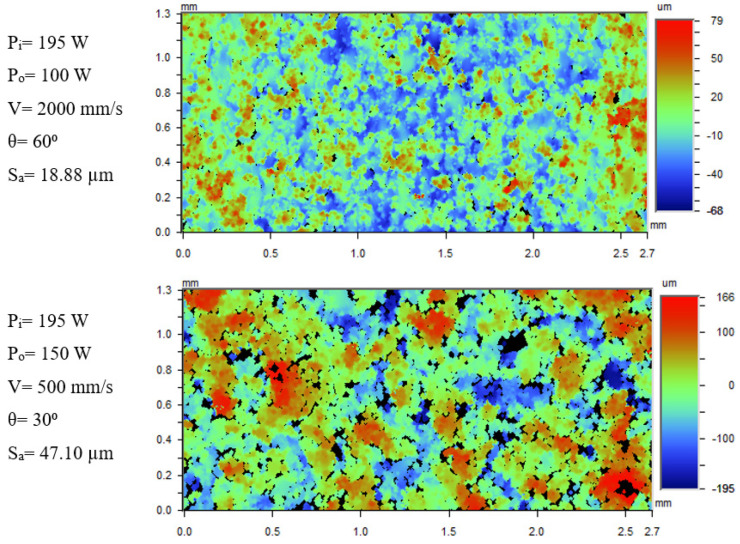
Downskin surface images of samples with lowest and highest average surface roughness.

The WLI images of the samples with the lowest and highest downskin surface roughness are shown in Figure 13. The sample with the highest downskin surface roughness (P_i_ = 195 W, P_o_ = 150 W, V = 500 mm/s, θ = 30°) has a large quantity of powder particles attached to it compared to the sample with lowest S_a_ (P_i_ = 195 W, P_o_ = 100 W, V = 2000 mm/s, θ = 60°). The percentage area of particles data in Figure 12 supports the image analysis. The strategy of using a higher LED inner contour and lower LED outer contour seems to be effective for reducing surface roughness at higher scan speeds.

**Figure 14:**
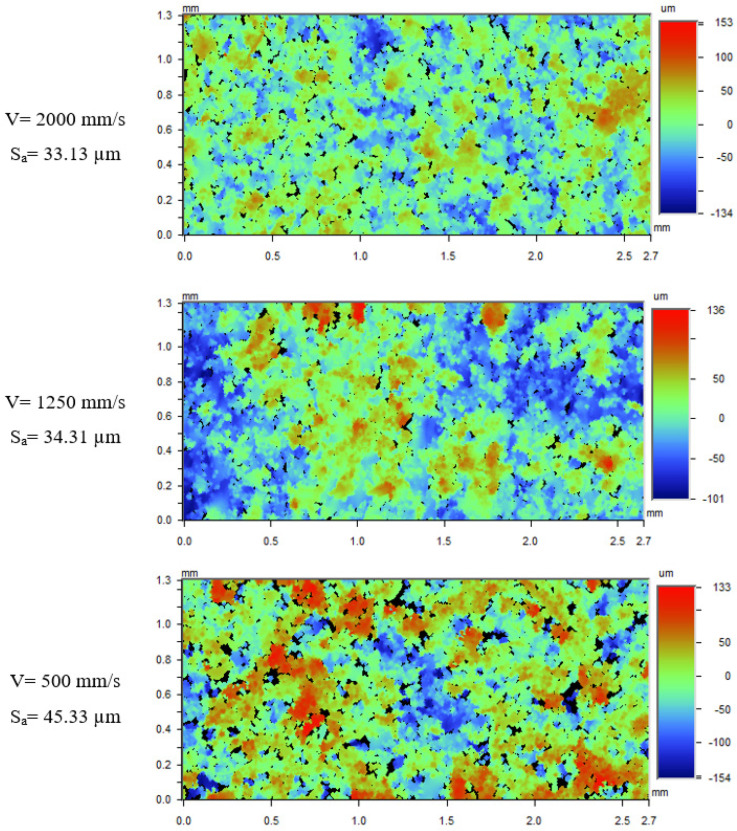
Downskin surface images of samples built with P_i_ = 195 W, P_o_ = 150 W, and θ = 30°, but different scan speeds.

Images of the downskin surface of 30°-inclined samples built with the same set of inner and outer contour scan powers (P_i_ = 195 W, P_o_ = 150 W) but different scan speeds are shown in Figure 14. There is a large quantity of powder particles present on the sample built with a scan speed of 500 mm/s compared to that with scan speed of 2000 mm/s. The smaller scan speed creates a larger melt pool due to increased time of laser exposure and hence has a higher surface area of melt pool exposed to the powder bed underneath the scan domain [27].

## 4. Discussion

The study reveals that both pre- and post-contouring strategies resulted in similar trends of surface roughness with the inclination angles and process variables. In pre-contoured samples, the lowest surface roughness of 14.08 µm was obtained for the 60°-inclined sample built with a low laser power (100 W) and high scan speed (2000 mm/s). The highest S_a_ of 42.84 µm was for the 30°-inclined sample with high LED (P = 195 W, V = 500 mm/s). The offset distance did not have a significant effect on the downskin surface roughness due to continuous remelting of the contouring region. Post-contouring using high LED for the inner contour and low LED for the outer contour, combined with a high scan speed, resulted in the low S_a_ of 18.88 µm for samples with a 60° inclination.

Irrespective of the strategy used, the downskin surface roughness is low at 30° inclination and it increases significantly for 45°- and 60°-inclined surfaces. The overhang distance between each layer is high for the 30°-inclined samples (51.9 µm) compared to the 45°- (30 µm) and 60° (17.3 µm)-inclined samples according to an equation proposed by Wang et al. (Equation (1)) [28].

S = H. cot θ(1)

Here, S is the overhang length between two layers, H is the layer thickness, and θ is the inclination angle [28]. The exposed melt pool region on the overhang causes dross formation and increases the surface area for partially melted powder particles. The surface skewness results showed that the peaks and valleys on downskin surfaces are not only process-induced but also due to random particle attachment (Figure 5 and Figure 11). In addition, the 30°-inclined surfaces showed a larger percentage area covered by particles (Figure 8 and Figure 12). The results align with Covarrubias et al.’s study on the effect of build angle on overhang surface quality. They also observed that the overhang surfaces built at smaller inclination angles have more visible partially melted particles, causing an increase in roughness [13]. 

Likewise, the downskin surface roughness is high at pre-contouring with high laser power and low scan speed (high-LED). At high LED, the melt pool dimensions are higher than low-LED cases [25]. This creates a larger melt pool area to be exposed to the powder bed underneath, aiding the attachment of partially melted particles to the surface. For post-contouring strategy, when both inner and outer contour scans had high laser power (P_i_ = 195 W, P_o_ = 150 W), the S_a_ value was higher too due to the large melt pool dimensions at high laser power. However, out of all the cases examined, the downskin surface roughness is lower when the outer contour laser power is low (100 W) and scan speed is high (2000 mm/s). 

While comparing the surface roughness of samples built with similar contouring process parameters and same slope, the pre-contoured samples have lower roughness, even though the difference is merely ~5 µm. The selection of contouring strategies and processing conditions hence need to be tailored according to the desired end-use application. The effect of these strategies on other surfaces like vertical or upskin surfaces is another factor to be considered.

**Figure 15:**
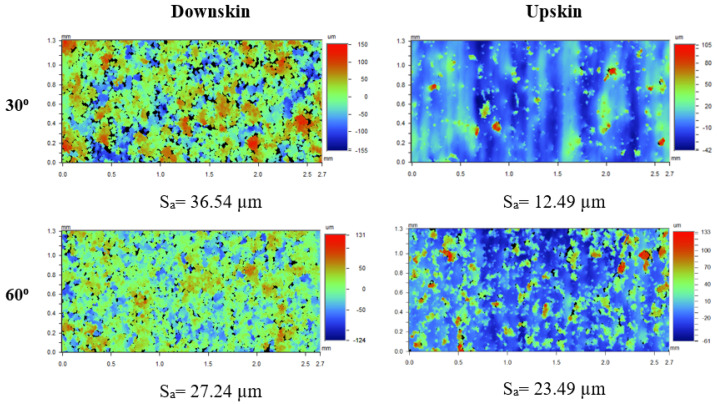
Downskin and upskin images of post-contoured samples built with P_i_ = 150 W, P_o_ = 100 W, V = 500 mm/s, and different inclination angles.

As an extension to this research, the upskin surfaces of fabricated samples were also analyzed [29]. The effect of scan strategy and contouring parameters on the downskin surfaces of both pre- and post-contoured samples are quite distinct from their respective upskin surfaces. Figure 15 compares the downskin and upskin surfaces of a post-contoured sample built with the medium-LED case (P_i_ = 150 W, P_o_ = 100 W, V = 500 mm/s) at low and high inclination angles. The upskin surface roughness is lowest for the 30°-inclined samples and it further increases with an increase in inclination angle. The upskin surfaces are mostly formed by the step edges, and the large distance between the step edges on 30°-inclined samples reduces their surface roughness [25]. While using the higher scan speed and low laser power aids in reducing the downskin surface roughness, it negatively impacts the finish of the upskin region. A larger melt pool created with a low scan speed and high laser power helps the remelting on upskin surface and reduces the surface irregularities. Also, compared to the upskin regions where remelting happens on the previously solidified bulk regions, the melt pool size on the downskin surface will be larger for the same input energy density [19]. The reason is the considerably lower heat conduction in the powder bed than the bulk regions because of the increased absorption of energy by powder particles during laser interaction [26].

## 5. Conclusions

This experimental study evaluated the effects of contouring scan strategies and the process parameters on the downskin surfaces of sloped L-PBF parts. The influence of pre- and post-contouring strategies with a wide range of processing conditions like laser power, scan speed, offset distance, and inclination angle were analyzed. The WLI images and the average surface roughness, S_a_, and surface skewness, S_sk_, were used for qualitative and quantitative analysis. The major conclusions from the study are listed below.

- Pre-contouring resulted in the lowest surface roughness (14.08 µm) for the 60°-inclined sample built with a low laser power (100 W) and high scan speed (2000 mm/s).
- Post-contouring with a higher LED inner contour scan and low-LED outer contour scan was effective in reducing the roughness at higher scan speeds. The sample built with 195 W inner contour laser power, 100 W outer contour laser power, and 2000 mm/s scan speed has the lowest S_a_ value of 18.88 µm.
- The pre-contouring at low LED resulted in overall smoother surface, but the difference in S_a_ values is very small (~5 µm) when samples built with similar conditions with both strategies are compared.
- In general, a lower LED at the downskin surface–powder bed interface resulted in lower surface roughness, irrespective of the contour scanning strategy. The heat conduction through the powder bed is lower compared to the solidified bulk regions, and hence low heat input will help in smaller melt pool formation. This aids in reducing the powder particle attachment to the exposed area of melt pool.
- The 30°-inclined samples have significantly higher downskin surface roughness compared to the 45°- and 60°-inclined samples. The overhang length is higher at smaller inclination angles, which results in more powder particle attachment to the downskin region.
- The conditions favorable for the lower downskin surface roughness have a negative impact on the corresponding upskin surfaces. A high LED helps in remelting the upskin surface which results in better surface finish. Also, the upskin surface roughness reduces with an increase in distance between step edges, and hence 30°-inclined samples have smoother surfaces.

The contouring of the scan domain is an effective method to reduce the downskin surface roughness of L-PBF parts. The selection of contouring scan strategy and processing conditions depends on the end-use application. However, this study provides a comprehensive approach to evaluating the process variables to tailor the downskin surface roughness of L-PBF parts. The careful consideration of contour scanning strategy and processing conditions can help in attaining a desired surface quality of the parts by eliminating the post-processing stages.

## Figures and Tables

**Table 1:** DOE with pre-contouring strategy.

Parameter No.	Laser Power, P (W)	Scan Speed, V (mm/s)	Offset Distance, d3 (µm)
1	100	500	20
2	100	1250	80
3	100	2000	120
4	150	500	120
5	150	1250	20
6	150	2000	80
7	195	500	80
8	195	1250	120
9	195	2000	20

**Table 2:** DOE with post-contouring strategy.

Parameter No.	Laser Power of Inner Contour Scan, P_i_ (W)	Laser Power of Outer Contour Scan, P_o_ (W)	Scan Speed, V (mm/s)
1	195	150	500
2	195	150	1250
3	195	150	2000
4	150	100	500
5	150	100	1250
6	150	100	2000
7	195	100	500
8	195	100	1250
9	195	100	2000

**Table 3:** ANOVA results of S_a_ data of pre-contoured samples.

Source	DF	Adj SS	Adj MS	F-Value	*p*-Value	Percent Contribution
P (W)	2	223.66	111.83	5.71	0.005	3.575
V (mm/s)	2	1078.8	539.41	27.5	0.000	17.25
d3 (µm)	2	83.760	41.880	2.14	0.125	1.339
θ (°)	2	3458.4	1729.2	88.3	0.000	55.29

**Table 4:** ANOVA results of S_sk_ data of pre-contoured samples.

Source	DF	Adj SS	Adj MS	F-Value	*p*-Value	Percent Contribution
P (W)	2	0.1586	0.0793	0.66	0.518	1.670
V (mm/s)	2	0.0647	0.0324	0.27	0.764	0.682
d3 (µm)	2	0.5768	0.2884	2.41	0.097	6.072
θ (°)	2	0.0831	0.0416	0.35	0.708	0.875

**Table 5:** ANOVA results of S_a_ data of post-contoured samples.

Source	DF	Adj SS	Adj MS	F-Value	*p*-Value	Percent Contribution
P_i_ (W)	1	1.0700	1.0700	0.06	0.802	0.023
P_o_ (W)	1	67.540	67.540	4.01	0.049	1.432
V (mm/s)	2	588.76	294.38	17.5	0.000	12.49
θ (°)	2	2777.4	1388.7	82.4	0.000	58.89

**Table 6:** ANOVA results of Ssk data of post-contoured samples.

Source	DF	Adj SS	Adj MS	F-Value	*p*-Value	Percent Contribution
P_i_ (W)	1	0.0904	0.0904	1.42	0.237	1.344
P_o_ (W)	1	0.2128	0.2128	3.34	0.072	3.164
V (mm/s)	2	0.3580	0.1790	2.81	0.067	5.322
θ (°)	2	1.4360	0.7180	11.3	0.000	21.35

## Data Availability

Data are contained within the article.
